# Complete Genome Sequence of *Bacillus bombysepticus*, a Pathogen Leading to *Bombyx mori* Black Chest Septicemia

**DOI:** 10.1128/genomeA.00312-14

**Published:** 2014-05-15

**Authors:** Tingcai Cheng, Ping Lin, Shengkai Jin, Yuqian Wu, Bohua Fu, Renwen Long, Duolian Liu, Youbing Guo, Li Peng, Qingyou Xia

**Affiliations:** State Key Laboratory of Silkworm Genome Biology, Southwest University, Chongqing, China

## Abstract

*Bacillus bombysepticus* is a Gram-positive spore-forming bacterium. Here, we announce the first complete genome sequence of this organism isolated from the cadavers of silkworm larvae that had been sick. The genome contains a single circular chromosome and a circular plasmid. Analyses of the *B. bombysepticus* genome will provide insights into its pathomechanisms and biology.

## GENOME ANNOUNCEMENT

*Bacillus bombysepticus* is a Gram-positive bacterium first identified by Hartman (1) from the corpses of silkworms (*Bombyx mori*) that had been sick. Infection with *B. bombysepticus*, a main bacterial pathogen of the silkworm, leads to the classical symptoms of black chest septicemia: a peutz appears on the thoracoabdominal region or the 1st to 3rd segment of the abdomen and then expands to the whole body. Previous experiments demonstrated that many genes and pathways of the silkworm are modulated after *B. bombysepticus* infection (2, 3). However, little is known about the pathogenesis of *B. bombysepticus* (4). A phylogenetic tree based on 16S rRNA sequences showed that *B. bombysepticus* is close to *Bacillus cereus* and *Bacillus thuringiensis* (2). Like *B. thuringiensis*, *B. bombysepticus* also produces spores and parasporal crystals and has a strong toxicity for silkworm larvae (2). Here, we sequenced the genome of *B. bombysepticus* in order to find the key genes of pathogenic infection and clarify the relationship between pathogen and host.

Genomic DNA of *B. bombysepticus* was extracted utilizing the E.Z.N.A. bacterial DNA kit (Omega). The genome was sequenced using high-throughput Solexa paired-end sequencing technology at the Beijing Genomics Institute (BGI) (Shenzhen, China). Filtered paired-end reads (2,116 Mb in total) were obtained, giving 363.6-fold coverage of the genome. The sequence reads were assembled into one chromosome and a plasmid using the SOAP*denovo* alignment tool (http://soap.genomics.org.cn/soapdenovo.html). The chromosome contains one large scaffold, including 2 contigs. Scaffolds were constructed with an *N*_50_ value of 5,296,309. All the intrascaffold and interscaffold gaps were then closed by sequencing of PCR amplification. SOAPaligner/SOAP2 (http://soap.genomics.org.cn/soapaligner.html) was used for sequence quality assessment.

The 5.87-Mb genome of *B. bombysepticus* contains two replicons, a circular chromosome (5,295,783 bp) and a circular plasmid (577,809 bp) named pBb. The G+C content of the chromosome is 35.25%, while that of the plasmid is 32.01%. The total numbers of genes and coding sequences (CDSs) identified are 5,702 and 5,488, respectively. Altogether, the genes were analyzed by using the databases of KEGG, COG, Swiss-Prot, TrEMBL, NR, and GO for function annotation. A total of 94 tRNAs and 39 copies of rRNA were identified by tRNAscan and RNAmmer, respectively. Also, the genome contains prophages, genomic islands (GIs), and two clustered regularly interspaced short palindromic repeats (CRISPRs). By the synteny analysis of amino acids and nucleotides between *B. bombysepticus* and *B. thuringiensis* serovar Chinensis CT-43, 4,488 genes were aligned, with an identity up to >96.49%, indicating that *B. bombysepticus* and *B. thuringiensis* have a common ancestor in the evolutionary process.

This is the first genome sequence of the *B. bombysepticus* species. The result will not only facilitate a better understanding of the physiology, metabolic potential, and virulence mechanisms of this pathogen but also open new perspectives on the functional genomics and the development of more efficient pest control strategies.

### Nucleotide sequence accession numbers.

The genome sequence of *B. bombysepticus* has been deposited at GenBank under accession no. CP007512 (chromosome) and CP007513 (plasmid pBb).
